# Testicular torsion: a modified surgical technique for immediate intravaginal testicular prosthesis implant

**DOI:** 10.1590/S1677-5538.IBJU.2021.9917

**Published:** 2021-08-05

**Authors:** Daniel Hampl, Leandro Koifman, Ricardo de Almeida, Marcio Ginsberg, Francisco J. B. Sampaio, Luciano A. Favorito

**Affiliations:** 1 Hospital Municipal Souza Aguiar Serviço de Urologia Rio de JaneiroRJ Brasil Serviço de Urologia, Hospital Municipal Souza Aguiar, Rio de Janeiro, RJ, Brasil; 2 Universidade do Estado do Rio de Janeiro Unidade de Pesquisa Urogenital Rio de JaneiroRJ Brasil Unidade de Pesquisa Urogenital, Universidade do Estado do Rio de Janeiro – Uerj, Rio de Janeiro, RJ, Brasil

**Keywords:** Spermatic Cord Torsion, Orchiectomy, Testicular Diseases

## Abstract

**Purpose:**

The aim of this paper is to propose a modified surgical technique for immediate intravaginal prosthesis implantation in patients undergoing orchiectomy due to testicular torsion, and to evaluate the wound healing process and patient’s satisfaction.

**Material and methods:**

We prospectively analyzed 137 patients with testicular torsion admitted to our facility between April 2018 and May 2020. Twenty-five patients who underwent orchiectomy were included in this study. Fifteen had a testicular prosthesis implanted at the same time as orchiectomy using a modified intravaginal technique (summary figure) and 10 received implants 6 to 12 months after orchiectomy. Wound healing was evaluated at a minimum of four checkpoints (on days 15, 45, 90 and 180 after surgery). At the end of the study, a questionnaire was administered to measure patients’ satisfaction rate. Student’s t test was used for comparison of quantitative data between negative vs. positive cultures (p <0.05). The chi-square test was used to verify associations between categorical variables and immediate vs. late prosthesis implantation (p <0.05).

**Results:**

Patient’s ages ranged from 13 to 23 years (mean 16.44 years). Overall time lapse from symptoms to orchiectomy ranged from 10 hours to 25 days (mean 7.92 days). Only one extrusion occurred and it happened in the late implant group. All wounds were healed in 72%, 88%, 95.8% and 100% of the cases on the 15th, 45th, 90th and 180th days after implant, respectively. At the end of the study, all patients stated they would recommend it to a friend or relative. The only patient that had prothesis extrusion asked to have it implanted again.

**Conclusion:**

There was no prosthesis extrusion using the modified intravaginal surgical technique for immediate testicular prosthesis implantation, which proved to be an easily performed and safe procedure that can avoid further reconstructive surgery in patients whose testicle was removed due to testicular torsion.

## INTRODUCTION

Testicular torsion (TT) affects 8.6 per 100.000 males per year between 16 and 25 years of age in the United States. It is considered a urological emergency that requires surgical management (1). Prompt surgical exploration is associated with greater salvage rates (2). Even in developed countries, one-third of testes are considered unsalvageable, thus requiring orchiectomy (3). Presentation delay, misdiagnosis and inter-hospital transfer time are the main factors that contribute to this tragic statistic (4).

TT can occur any time during a man’s life, but is more frequent in adolescents (1). The absence of a testicle in adolescents, who are particularly sensitive to negative body image, sociocultural influences and social comparison, can lead to a severe feeling of unhappiness with appearance (5, 6).

Although immediate prosthesis implant is an option for esthetic reconstruction in the emergency setting, it has been largely avoided, especially in the case of late TT surgical exploration, presumably because of the increased complication rate (7, 8).

Recently, some authors, in line with new advances in testicular prosthesis manufacture, have revisited this question (9). The evidence is, however, limited, since most human studies are small case-series of testicular torsion as the only reason for device implantation.

Our hypothesis was that the tunica vaginalis (TV) is a covering layer that can help to avoid testicular prosthesis extrusion. The aim of this paper is to propose a modified surgical technique for the immediate intravaginal prosthesis implantation in patients whose unsalvageable testicle is removed due to testicular torsion. Besides that, we evaluated the scrotum wound healing process and patient’s satisfaction with the implants.

## MATERIAL AND METHODS

This study received institutional review committee approval (IRB number 04411118.1.0000.5279) and was carried out in accordance with the ethical standards of the hospital’s institutional committee on human experimentation.

We prospectively analyzed 137 patients with testicular torsion admitted to our facility with diagnosis of testicular torsion between April 2018 and May 2020. We included patients aged 13 years or older, with stage III or higher on the Tanner Scale (10) of genital development, who underwent orchiectomy in response to testicular torsion.

Preoperatively, all patients were asked for informed consent regarding the option for and timing of prosthesis implant, after description of the surgical risks. Twenty-four patients decided not to be submitted to immediate implantation.

We excluded 31 patients whose testicular salvage was possible, 52 patients currently taking antibiotics or who had used any antibiotics up to 10 days before the procedure or taking medication on a regular basis for chronic or autoimmune diseases, 9 patients with high clinically suspicion of septic genital skin (combination of hyperemia, local heat and scrotal retraction), 14 patients that opted not to have the prosthesis implant at any time and six patients whose parents or legal guardians decided not to sign the informed consent form.

After these exclusions, 25 patients were included in the study and operations were performed by the same surgeon. Fifteen patients had an immediate prosthesis implant with a modified surgical technique at the time of orchiectomy. We contacted the 24 patients who decided not to have the immediate implant performed and ten of them were included in the late implant group.

In the operating room (OR), a single dose of cefazolin (2g) was given as a systemic prophylactic antibiotic against Gram-positive and Gram-negative bacteria. The external genitalia were shaved to remove hair from the surgical site.

We routinely performed surgical explorations using two separate transverse scrotal incisions. Following the orchiectomy in cases of non-viable testicles, the spermatic cord stump was ligated with two 2-0 cotton hemostatic sutures. The tunica dartos was closed with a running absorbable monofilament 4-0 suture and the skin was closed with separate nylon 4-0 stitches. We routinely performed contralateral orchiopexy.

Silimed^®^ made available, for the study, the elastomer version of its silicone-gel filled prosthesis with three different volumes (10cc, 20cc and 30cc). The ideal implant volume was chosen using an orchidometer to estimate the volume of the healthy testicle. No antibiotic solution was used to irrigate the wound or implant.

Late testicular prosthesis implantations were performed using an inguinal incision. Using finger dissection, a blunt subdartos dissection was performed to allow mobilization and to create space for the implant accommodation. By inverting the most pendent part of the scrotum, the prosthesis was anchored to the dartos tunica with a nylon 4-0 suture. We did not put additional sutures cephalic to the prosthesis to prevent its migration. Scarpa’s fascia was closed with running absorbable monofilament 3-0 suture and the skin was closed with a subdermal running suture of nylon 2-0 which was removed on the first follow up consult.

**Figure 1:**
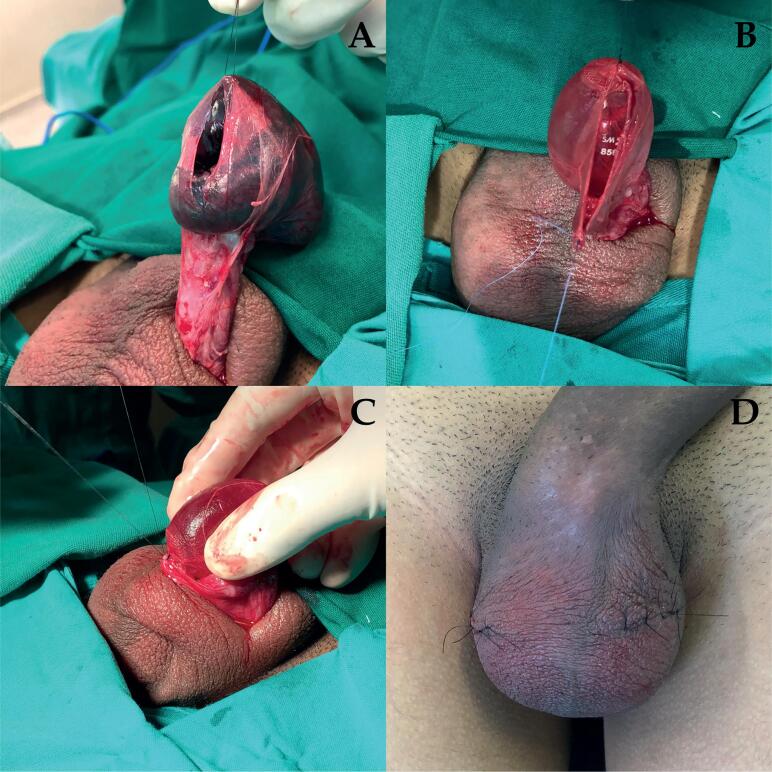
Immediate prosthesis implantation in a 16 years-old boy using the proposed surgical technique. A) TV mobilization and posterior incision to the tunica vaginalis cavity, B) Prosthesis placed and sutured to the most pendent part of the TV cavity, C) Prosthesis anchored at the most pendent part of the scrotum to the dartos tunica, D) Immediate post-operative in the OR.

The immediate testicular prosthesis technique (Figure-1) was performed with the same preoperative care in the OR. We also used a bilateral scrotal incision, but on the torced side the incision was 2cm higher than on the contralateral side. With fingers, a blunt subdartos dissection was performed to allow mobilization of the TV. A nylon stitch was placed to expose the posterior wall of the TV cavity, where it was incised to expose the testicle and the torsed spermatic cord. Before proceeding with orchiectomy and implant handling, the gloves of the surgical team were changed. Following the orchiectomy of patients with non-viable testicle, the spermatic cord stump was ligated with two 2-0 cotton hemostatic sutures. The testicular implant was placed inside the cavity and anchored to the TV with a nylon 3-0 stich. This same nylon thread, after closure of the TV with running absorbable monofilament 3-0, was used to anchor the implant to the dartos tunica at the most pendent part of the scrotum. The tunica dartos was closed with a running absorbable monofilament 4-0 suture and the skin with a separate nylon 3-0 suture. This way, the TV incision was placed posteriorly and there was no contact with the anterior wall of the scrotum where the dartos and skin incisions were made. With this technique we tried to avoid overlapping incisions.

All patients were discharged in the first 24 hours after surgery and cephalexin was prescribed for five days. Follow-up with the same urologist included a minimum of four checkpoints on days 15, 45, 90 and 180 after the surgical procedure. The modified Southampton Wound Score System (11) (mSWSS) was used to evaluate wound healing (Supplemental File-1).

On day 180, all participants filled in a questionnaire about their satisfaction with the implant. The questionnaire consisted of scoring from 0 (very bad) to 4 (very good) aspects such as device position, consistency, size, wound healing and whether the patient would recommend prosthesis implantation to a friend or relative suffering the same TT condition.

All parameters were statistically processed and tabulated. The Student t-test was used for comparison of quantitative data between late vs. immediate implant (p <0.05). The chi-square test was used to verify associations between categorical variables and late vs. immediate implant (p <0.05). The statistical analysis was performed with the IBM SPSS program (Version 20).

## RESULTS

We analyzed 25 men with unsalvageable testicular torsion who successfully underwent testicular prosthesis implantation with different timing, with a median follow-up of 18.16 months (range 8.16 to 28.2).

Patients ages ranged from 13 to 23 years (mean age 16.44±3.31 years) (Table-1). There was no difference between groups considering time lapse from symptoms to orchiectomy (mean 7.92 days, p=0.217), side affected (p=0.211) or hydrocele presence at the time of orchiectomy (p=0.667). Sixteen patients had medially twisted testicle while 9 had lateral twisting (p=0.691).

**Table 1:** Differences in perioperative characteristics and postoperative outcomes between immediate and late testicular prosthesis insertion.

		Time of implant			
		Late	Immediate	Total	p-value
Age (median±sd)		16.00±3.712	16.73±3.127	16.44±3.318	0.599^b^
**Side, n (%)**					
	Left	6	4	10	0.211^a^
	Right	4	11	15	
**Hydrocele, n (%)**					
	No	4	4	8	0.874^a^
	Yes	6	11	17	
**Twist direction, n (%)**					
	Medial	7	9	16	0.691^a^
	Lateral	3	6	9	
Time from pain to orchiectomy (in days) (median±sd)		6.16±4.407	9.09±6.328	7.92±5.726	0.217^b^
Time from orchiectomy to implant (in days) (median±sd)		265.94±76.330	0		-
**mSSWS 15 days after implantation, n (%)**					
	Normal healed	9	9	18	0.179^a^
	Minor complication	1	6	7	
**mSSWS 45 days after implantation, n (%)**					
	Normal healed	10	12	22	0.250^a^
	Minor complication		3	3	
**mSSWS 90 days after implantation, n (%)**					
	Normal healed	9	14	23	1.000^a^
	Minor complication		1	1	
**mSSWS 180 days after implantation, n (%)**					
	Normal healed	9	15	24	-

**Sd** - standard deviation

a chi-square test

b Student t-test

The mSWSS revealed normal wound healing in 60% on day 15 after the immediate implant, while 6 patients had minor complications, compared to only 1 patient who had minor complication in the late group (p=0.174). Minor complications were treated by optimizing the local hygiene.

On day 45 after the implantation, all patients in the late group had uncomplicated fully healed wounds while 20% of patients in the immediate group had minor complications (p=1.000).

One patient, who had late implant performed through an inguinal incision 257 days after orchiectomy, showed normal inguinal incision healing on day 45 but extruded the implant through the previous scrotal scar 60 days after implantation. The implant removal was performed under local anesthesia as an outpatient and oral administration was prescribed for 7 days of amoxicillin-clavulanate. This patient was removed from the statistical analyses due to extrusion.

On day 90, one patient in the immediate implant group demonstrated some skin erythema at one point (minor complication), with no need for intervention.

On day 180, all patients were healed and filled in a questionnaire to evaluate if they were satisfied and if they would recommend the implant. Implant position and size were considered very good by 79.2% and 83.3% of patients respectively (Table-2). Consistency was considered very good by all patients in the late group while only 53.3% of patients in the immediate group shared this opinion (p=0.052). Final aspect of the scar was considered very good in 77.8% and 93.3% of patients in the late and the immediate groups, respectively. Irrespective of implant timing all patients stated they would recommend it to a friend or relative.

**Table 2:** Patient’s satisfaction with the implant.

		Time of implant			
		Late	Immediate	Total	p-Value
**Volume, n (%)**					
	10cc				
	20cc	9	15	24	
	30cc				
**Position, n (%)**					
	Very bad				
	Bad				
	Indiferent				
	Good	2	3		1.000^a^
	Very good	7	12		
**Consistency, n (%)**					
	Very bad				
	Bad				
	Indifferent		1		
	Good		6		0.052^a^
	Very good	9	8		
**Size, n (%)**					
	Very bad				
	Bad				
	Indifferent				
	Good		4		0.259^a^
	Very good	9	11		
**Scar, n (%)**					
	Very bad				
	Bad				
Indifferent					
	Good	2	1		0,533^a^
	Very good	7	14		
**Would recommend, n (%)**					
	No				
	Yes	9	15		
Follow-up, days (median±sd)		597.8±42.56	512.5±169.53	544.5±141.08	0.081^b^

a chi-square test

b Student t-test.

## DISCUSSION

Undescended testicle and testicular atrophy are the most common conditions where prostheses are implanted. Interestingly, the main cause of orchiectomy in men from 0 to 25 years old is testicular torsion (12). It is estimated that less than 25% of testicular prostheses are placed in response to torsion (13).

Complications as infection and extrusion of the prosthetic device are clearly feared by surgeons (14, 15). But we should consider the fact that even when these complications occur, this will not be a life-threatening situation (13, 16).

Considering TT is an inflammatory/infectious condition, previous studies have reported that patients are more susceptible to complications such as infections and extrusion (7). Because of that, some authors have suggested that testicular prosthesis implant should be performed between 6 to 12 months after orchiectomy (17, 18).

On the other hand, a recent study clouded this issue by suggesting that the vast majority of the TV cavity remains aseptic in cases of testicular torsion even when reactive hydrocele is present (19).

Furthermore, testicular prosthesis implant can lead to a significant improvement in body image (20). It can also improve self-satisfaction, self-esteem, physical attractiveness and positive feelings during sexual activity (21).

Recently, many authors have tried to define the best timing of testicular prosthesis implantation as reconstructive surgery in patients suffering from absence of a testicle (9, 18, 22, 23). In 2012, Bush and Bagrodia (9) reported good initial results of combined orchiectomy and prosthesis exchange in 12 patients treated for testicular torsion with follow-up from 1.5 to 16 months. Because of the intravaginal approach, their technique closely resembles ours, but we think that mobilization of the TV should always be performed to allow a posterior incision in the TV to avoid overlapping incisions. When there are no overlapping suture lines, the extrusion process might be hindered.

Considering that scrotal and dartos layers are embryologically distinct from the other internal layers of the scrotum wall, and they also have their own blood and nerve supplies, it is very unlikely for them to share the same infectious/necrotic process. Therefore, we aimed to improve this intravaginal testicular prosthesis implant technique by trying to maintain the natural integrity of existing tissue.

It is known that regular skin flora is the main source of infection of prosthesis sites (14) and TV mobilization can prevent mishandling the implant and its accidental contact with the skin. Another characteristic of the technique is to maintain the cremasteric reflex preserved by keeping as many cremaster fibers as possible.

Consistency of the implant is the most common complaint about testicular prostheses (24). Although there was no statistical difference, in our study this opinion was more common in the immediate group. On the other hand, final scar aspect was more criticized by patients in the late group. Perhaps immediate exchange of the testicle for the implant highlights consistency disparity between the implant and natural testicle. On the other hand, having two different scars is what most bothered the patients in the late group.

Although we cannot make a categorical statement, we stress there was no extrusion in the immediate implant group. Perhaps a larger sample could statistically confirm that the intravaginal technique is secure and should be considered the first-line treatment for patients submitted to orchiectomy as part of their treatment for testicular torsion.

This study has many limitations. The small sample is an evident drawback, but the study was interrupted by the Covid-19 pandemic. Also, there was no culture sampling of the tunica vaginalis cavity, which could be important to guide antibiotic treatment in patients that showed complications during follow-up. Since there was only one surgeon conducting the operations, the feasibility of the technique has not been sufficiently tested yet and there was no comparison of clinical analyses during follow-up.

## CONCLUSIONS

Based on these findings, we are of the opinion that there is no impediment to immediate prosthesis implantation in the testicular torsion setting, especially in cases with late presentation when there is no doubt about the testis viability.

## ABBREVIATIONS

mSWSS: Modified Southampton Wound Score System
OR: Operating room
TT: Testicular torsion
TV: Tunica vaginalis

## APPENDIX

**Supplemental File 1:** This table reports the results of the modified Southampton Scoring System used to analyze our sample during the follow-up checkpoints.

	Southampton Scoring System	
	Score	Appearance
**Normal healing**	0	Normal healing
	1	Normal healing with mild bruising or erythema
	A	Some bruising
	B	Considerable bruising
	C	Mild erythema
	2	Erythema plus other signs of inflammation
	A	At one point
	B	Around sutures
**Minor complication**	C	Along wound
	D	Around wound
	3	Clear or haemoserous discharge
	A	At one point only (<2cm)
	B	Along wound (>2cm)
	C	Large volume
	D	Prolonged (>3 days)
	4	Pus (antibiotic needed)
**Major complication**	A	At one point only (<2cm)
	b	Along wound (>2cm)
**Extrusion**	5	Deep or severe wound infection or Extrusion; (surgical approach needed); Implant removal
